# A network pharmacology-based approach to explore mechanism of action of medicinal herbs for alopecia treatment

**DOI:** 10.1038/s41598-022-06811-6

**Published:** 2022-02-18

**Authors:** Jungtae Leem, Wonmo Jung, Hi-Joon Park, Kyuseok Kim

**Affiliations:** 1 Research Center of Traditional Korean Medicine, Wonkwang University, 460, Iksan-daero, Sin-dong, Iksan, 54538 Jeollabuk-do Republic of Korea; 2 Acupuncture and Meridian Science Research Center, College of Korean Medicine, Kyung Hee University, Seoul, 02447 Republic of Korea; 3 Department of Science in Korean Medicine, Graduate School, Kyung Hee University Korean Medicine Hospital, Seoul, 02447 Republic of Korea; 4 Department of Ophthalmology, Otolaryngology, and Dermatology, College of Korean Medicine, Kyung Hee University, Seoul, 02447 Republic of Korea; 5 Department of Ophthalmology, Otolaryngology, and Dermatology, Kyung Hee University Korean Medicine Hospital, Seoul, 02447 Republic of Korea

**Keywords:** Computational biology and bioinformatics, Molecular biology, Skin diseases

## Abstract

Hair loss is one of the most common skin problems experienced by more than half of the world's population. In East Asia, medicinal herbs have been used widely in clinical practice to treat hair loss. Recent studies, including systematic literature reviews, indicate that medicinal herbs may demonstrate potential effects for hair loss treatment. In a previous study, we identified medical herbs used frequently for alopecia treatment. Herein, we explored the potential novel therapeutic mechanisms of 20 vital medicinal herbs for alopecia treatment that could distinguish them from known mechanisms of conventional drugs using network pharmacology analysis methods. We determined the herb-ingredient–target protein networks and ingredient-associated protein (gene)-associated pathway networks and calculated the weighted degree centrality to define the strength of the connections. Data showed that 20 vital medicinal herbs could exert therapeutic effects on alopecia mainly mediated via regulation of various target genes and proteins, including acetylcholinesterase (AChE), phospholipase A2 (PLA2) subtypes, ecto-5-nucleotidase (NTE5), folate receptor (FR), nicotinamide *N*-methyltransferase (NNMT), and quinolinate phosphoribosyltransferase (QPRT). Findings regarding target genes/proteins and pathways of medicinal herbs associated with alopecia treatment offer insights for further research to better understand the pathogenesis and therapeutic mechanism of medicinal herbs for alopecia treatment with traditional herbal medicine.

## Introduction

Approximately 50% of people experience hair loss throughout their life, which causes emotional and psychological problems and worsens the quality of life^1^. This may lead to infrequent social interaction, lack of confidence, and may affect self-esteem^2^. Although many therapeutic interventions have been developed and adopted for alopecia treatment, their preventive and therapeutic effects are not satisfactory^3^. Minoxidil (MXD) and finasteride drugs approved by the US Food and Drug Administration (FDA) for alopecia treatment exhibit limited efficacy and are associated with recurrent cases after cessation^4^. Side effects, such as scalp dryness, skin irritation (MXD), and sexual and psychological disorder development (finasterid), are limitations of conventional therapy^5^. Owing to this reason, many patients undergo complementary and alternative medicine (CAM) interventions^4^.

In East Asia, traditional herbal medicine (THM) is widely used for alopecia treatment in clinical practice^6,7^. In a recent retrospective observational study, 142 of the 222 patients (64%) presented with > 90% hair recovery following subjection to traditional Chinese medicine (TCM) treatment^6^. In a recent systematic review conducted considering 30 TCM randomized clinical trials, adjunctive TCM therapy demonstrated an increased total effective rate, improved microelement level, and decreased symptom score of refractory alopecia compared to the Western medication-only group^7^. In terms of safety, the therapy presented with a tendency of reduced adverse events in the meta-analysis (odds ratio, 0.55; 95% confidence interval, 0.29–1.05)^7^. Experimental studies conducted on alopecia have proposed several possible mechanisms of action of THM preparations, such as appropriate functioning of the hair growth cycle, inflammation, apoptosis, hormones, and angiogenesis^5^. Moreover, enhanced scalp blood circulation, nutritional support, and inhibition of 5α-reductase activity were reported as mechanisms mediating the activity of herbal preparations^8^. These studies indicate that THM may demonstrate potential therapeutic effects in alopecia treatment^6^.

However, system-level therapeutic mechanisms for combinations of medicinal herbs for alopecia treatment are not well established. Medicinal herbs may be more effective in alopecia associated with multiple pathogeneses because multiple components of the herbs affect multiple targets^5^. Network pharmacology is a novel approach to investigate the system-level mechanisms of medicine^9^, and the method incorporates *“the potential mechanism of multiple compounds*” and *“the pathways associated with the target of the compounds*”^10^. The core concept of network pharmacology is appropriate for multi-component and multi-targeted agents and is suitable for comprehensively exploring the complex mechanisms of THM^11^. The network pharmacology-based approach exhibits advantages in the discovery of active compounds and the potential mechanism of THM^12,13^. Therefore, network pharmacology is widely used to explore the therapeutic mechanism of THM, including the herb-compound-target network, target interpretation, and related biological functions and diseases^11,14,15^. In the present era, network pharmacologic approaches are utilized to provide insights into a molecular basis for the formulation of experience-based TCM theory and treatment strategy^12,13^.

In a previous study conducted on the exploration of the modular characteristics of medicinal herbs for alopecia treatment in TCM using network analysis, we identified medicinal herbs frequently used for alopecia treatment^16^. However, the therapeutic mechanism of hair loss by THM is not well established. In addition, we anticipate that the mechanism of hair loss treatment of THM might also have different mechanisms from that of conventional drugs. The present study aimed to determine the novel therapeutic mechanisms of action of THM preparations that could differentiate them from the conventional drugs for hair loss treatment.

## Methods

**Figure 1:**
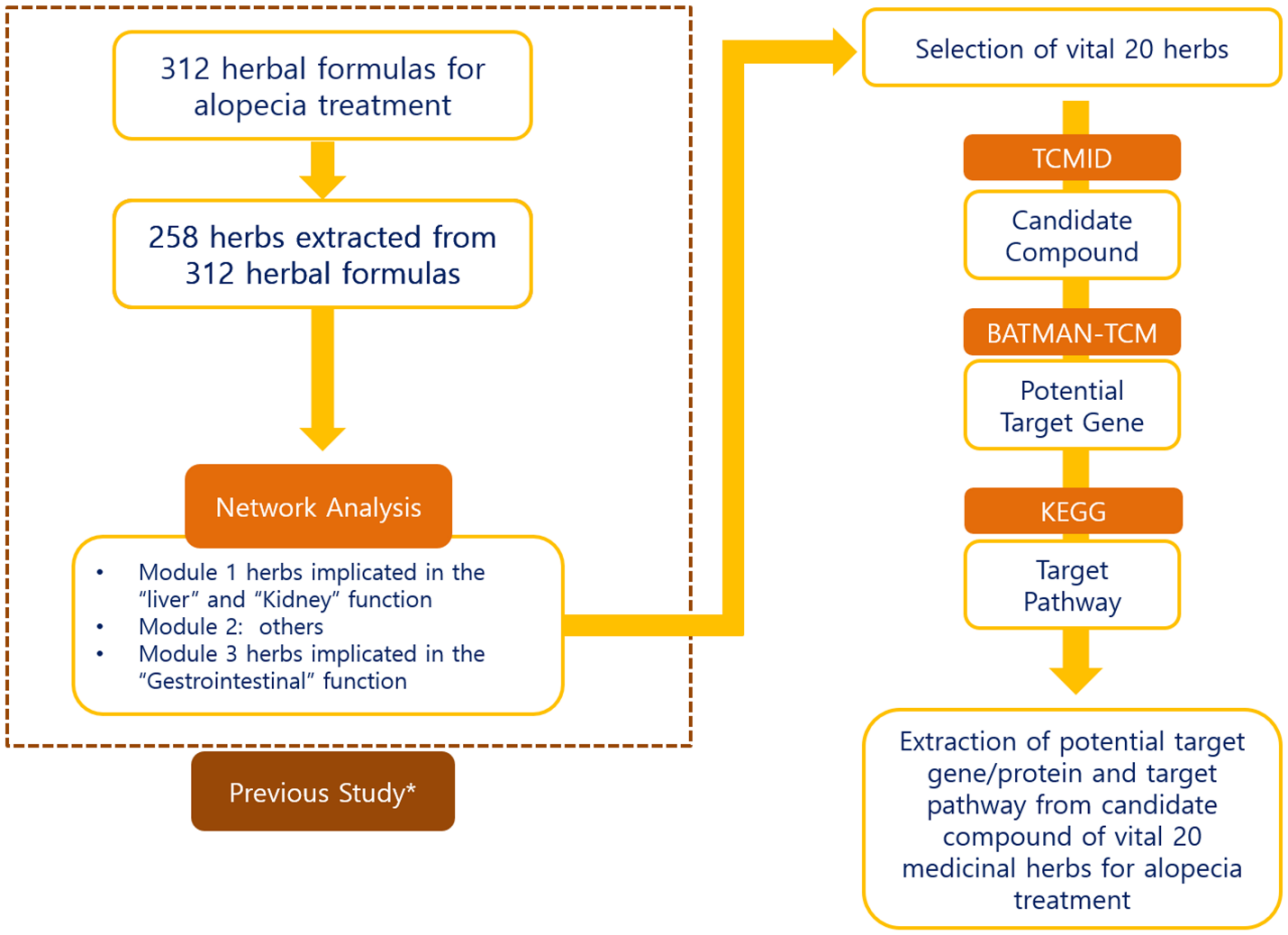
Schematic study workflow diagram**.** *TCM* traditional Chinese medicine, *TCMID* traditional Chinese medicine integrated database, *BATMAN* bioinformatics analysis tool for molecular mechanism, *KEGG* Kyoto encyclopedia of genes and genomes. Asterisk: Previous study: Leem et al. 2018^16^.

In this study, we explored the biological pathways of medicinal herbs for alopecia treatment using network pharmacologic analysis. The workflow of this study is presented in Fig. 1.

### Selection of medicinal herbs for alopecia treatment based on a previous study

In a previous network analysis study^16^, we investigated 312 herbal prescriptions for alopecia treatment, from which we derived and presented a network of 258 medicinal herbs via modularity analysis. Based on the analysis, we divided the 258 medicinal herbs into three modules (Module 1, 2, and 3). Those included in Module 1 were closely associated with the “Liver” and “Kidney” as per the TCM theory. Moreover, Module 2 herbs seemed to affect the body surface and were frequently used for external preparations, whereas Module 3 herbs were related to the “Stomach” (digestive function) as per the TCM theory. In the TCM theory, each medicinal herb in an herbal prescription can be categorized into the king, minister, assistant, or ambassador (in Chinese 君臣佐使, pronounced as Jun-Chen-Zuo-Shi) group, which act as major (king), complementary (minister), neutralizing (assistant), and delivery/retaining (ambassador) components, respectively^17^. Most Module 1 medicinal herbs were categorized into the “king” or “minister” group in herbal prescription for alopecia treatment. Therefore, we selected Module 1 herbs for further network pharmacologic analyses. Previously, research based on a network analysis (association rule mining) of the top 20 medicinal herbs in each module in the order of frequency was conducted. Based on the results obtained, we selected the top 20 medicinal herbs of Module 1 for further investigation as presented in Table 1. Detailed ingredients of Module 1 medicinal herbs are provided in Supplementary Material 1.

**Table 1:** List of the top 20 main medicinal herbs in Module 1 from a previous study.

Medicinal herbs in Module 1	Pinyin in the TCMID database
*Polygonum multiflorum* Thunb. (何首烏)	HE SHOU WU
*Angelica sinensis* (Oliv.) Dlels (當歸)	DANG GUI (CHAO XIAN DANG GUI)
*Rehmannia glutinosa* Libosch. (Prepared) (熟地黃)	SHU DI HUANG
*Ligusticum chuanxiong* Hort. (川芎)	CHUAN XIONG
*Ligustrum lucidum* Ait. (女貞子)	NU ZHEN ZI
*Eclipta prostrata* L. (旱蓮草)	MO HAN LIAN
*Lycium barbarum* L. (枸杞子)	GOU QI ZI
*Rehmannia glutinosa* Libosch. (生地黄)	SHENG DI HUANG
*Cuscuta chinensis* Lam. (菟絲子)	TU SI ZI
*Astragalus membranaceus* (Fisch.) Bge. (黃芪)	HUANG QI
*Paeonia lactiflora* Pall. (白芍藥)	BAI SHAO
*Salvia miltiorrhiza* Bge. (丹蔘)	DAN SHEN
*Morus alba* L. (桑椹)	SANG SHEN
*Sesamum indicum* L. (黑芝麻)	HEI ZHI MA
*Platycladus orientalis* (L.) Franco (側柏葉)	CE BAI YE
*Gastrodia elata* Bl. (天麻)	TIAN MA
*Carthamus tinctorius* L. (紅花)	HONG HUA
*Codonopsis pilosula* (Franch.) Nannf. (唐蔘)	DANG SHEN
*Chaenomeles speciosa* (Sweet) Nakai (木瓜)	MU GUA
*Polygonatum sibiricum* Red. (黃精)	HUANG JING

Related to the liver and kidney.

### Pharmacological network construction based on 20 vital medicinal herbs

To construct a tripartite network of compound (ingredient)-associated target gene-associated target pathway, we used data derived from the TCM Integrated Database (TCMID) and Bioinformatics Analysis Tool for Molecular Mechanism of TCM (BATMAN-TCM)^18,19^. Since, the TCMID includes data on 43,413 compounds derived from 8159 medicinal herbs, compound information was obtained from such a database^18^. We selected potential compounds based on oral bioavailability (OB ≥ 30) and drug-likeness index (DL ≥ 0.18) for integrative absorption, distribution, metabolism, and elimination (ADME) screening using default values obtained from the TCMID^18^. OB is an important pharmacokinetic index that is used to indicate the extent and speed of absorption into the blood circulation of orally administered drugs^15,18,19^, whereas DL aids estimation of the structural similarity between the related drug and ingredients of medicinal herbs. It also helps predict specific compounds that can be developed as drugs. Both OB and DL are utilized to perform screening of suitable compounds for drug development^11^. Since, BATMAN-TCM could provide target prediction information based on the existing drug-target interaction information, we used the target gene prediction score of compounds in 20 vital medicinal herbs based on the available data^19^. The drug prediction algorithm of BATMAN-TCM helps determine drug-drug similarity based on chemical structure and functional group based on similarity.

Protein–protein similarity was predicted by considering protein sequences and gene ontology (GO) analysis. The final prediction scores were calculated based on the similarity ranking. We defined a compound (ingredient) with a target gene prediction score of > 55 as a positive interaction. We only extracted data and utilized compound–target gene interactions that met the positive interaction criteria. Next, to analyze gene–pathway associations, pathway data derived from the Kyoto Encyclopedia of Genes and Genomes (KEGG) database was used, which highlighted 29,039 interactions of 325 human pathways related to 7373 human proteins^20^. KEGG helps provide target interpretation by highlighting high-level functions and molecular information for utilizing biological systems^11^. During this stage, PubChem ID was utilized as a unique gene ID that could be used to link extracted compounds whose information was derived from various databases^21^.

### Calculation of weighted degree of centrality

One of the purposes of network analysis is to investigate the important nodes in a graph structure. A variety of centrality measures can be used to define the strength of the connections between the individual nodes in the graph structure^22–24^. Classical degree centrality represents the simplest centrality measure and helps determine the number of direct connections of a specific node in the network. The advantage of degree centrality is the interpretability and comprehensibility of the results^25^. In this study, we evaluated the weighted degree centrality of individual associated pathways in a tripartite network consisting of compounds, target genes, and associated pathways. We presented only statistically significant weighted degree centralities compared to those obtained using the null model.

The weighted degree centrality of an associated pathway i (s_i_) is calculated as follows:

$${S}_{i}=\sum_{j=1, {n}_{1}}{w}_{ij}\times {e}_{ij}$$

$${w}_{ij}=\frac{\sum_{k=1,{n}_{2}}\sqrt{{p}_{jk}}\times {e}_{jk}}{\mathrm{ln}(\sum_{k=1,{n}_{2}}{e}_{jk})}$$

$${e}_{ij}=\left\{ {\begin{array}{ll} {1}& \quad {if \; edge \; exists \; between \; associated \; pathway \; i \; and \; target \; gene \; j}\\ {0}& \quad{otherwise} \end{array}} \right.$$

$${e}_{jk}=\left\{ {\begin{array}{ll} {1}& \quad {if \; edge \; exists \; between \; target \; gene\; j \; and \; compound \; k}\\ {0}& \quad{otherwise} \end{array}} \right.$$

$${p}_{jk}=prediction \; score \; between \; target \; gene \; j \; and \; compound \; k,$$

where n_1_ is the total number of target genes, n_2_ is the total number of compounds.

### Repeated construction of the null model for statistical comparison

For statistical comparison with the null model, we repeatedly generated random networks that were constructed by selecting 20 medicinal herbs randomly with a similar number of compounds derived from 8159 medicinal herbs registered in TCMID. Additionally, we constructed a tripartite network using data derived for 20 randomly selected medicinal herbs, in a manner similar to that adopted for the Module 1 herb tripartite network. From the random network, extraction of data on the weighed degree centralities as described in “Pharmacological network construction based on 20 vital medicinal herbs” and “Calculation of weighted degree of centrality” was performed. To select comparable medicinal herbs in terms of the number of included compounds, division of 8159 TCMID medicinal herbs into the following four bins according to the number of included compounds was performed as follows: bin 1, < 10; bin 2, 11–20; bin 3, 21–40; and bin 4, > 41 compounds.

We further matched the number of herbs in each bin and randomly selected medicinal herbs to construct a null model. The construction of a random network and the extraction of weighted degree centralities of associated pathways were repeated 10,000 times. Based on the cumulative weighted degree of centralities, we estimated the null model probability density of each associated pathway. Additionally, we conducted adjustment for multiple comparisons by lowering the false discovery rate (FDR) level to < 0.05, using the Benjamini–Hochberg procedure^26^. We finally discovered and highlighted associated pathways that showed a statistically significant weighted degree of centrality compared to that of the null model.

### Presentation of results

To better understand the analysis methods used, herb-compound-target gene network as a visualized network figure has been presented in this study. Data on the associated target genes and associated pathways of compounds in vital medicinal herbs, using the analysis described in “Pharmacological network construction based on 20 vital medicinal herbs” to “Repeated construction of the null model for statistical comparison” , were derived. The ingredients, related herbs, target genes, and associated pathway data are shown in Table 2. We also investigated the mechanisms of action of herbal medicines for treatment of alopecia, which have not been reported frequently in conventional medicine, and focused specifically on construction and visualization of compound–target gene–pathway networks. Furthermore, a tabular format for information transmission and readability rather than a figure format was adopted. Target proteins and associated pathways have been presented according to the number of compounds associated with the target protein (Table 3). When ‘alopecia’ was explored as a disease keyword in the GeneCards database, information regarding human genes, diseases, and pathways was obtained. Comparison of these results with our findings on target proteins of medicinal herbs for alopecia treatment has been presented in Table 3^27^. Table 4 shows the pathways associated with the 20 vital herbs based on the frequency of associated pathways identified in Table 3.

**Table 2:** List of 20 vital medicinal herb–ingredient–associated protein–associated pathways.

20 vital medicinal herbs	Ingredients	Associated target proteins	Associated target pathways
*Polygonum multiflorum* Thunb. (何首烏)	Polygodial	Acetylcholinesterase (AChE, Cartwright blood group)	Glycerophospholipid metabolism
*Angelica sinensis* (Oliv.) Dlels (當歸)	4-Ethylresorcinol, dihydropinosylvin, carvacrol, carvacrol acetate, dimethyl phthalate, *m*-cresol, *o*-cresol, *p*-cresol, *m*-ethylphenol, *p*-ethylphenol	AChE (Cartwright blood group)	Glycerophospholipid metabolism
	Azelaic acid, decanoic acid, hexadecanoic acid	Phospholipase A2 group IB (PLA2G1B)	Glycerophospholipid metabolism, ether lipid metabolism
	Dodecenoic acid, sebiferic acid	Lysophosphatidylcholine acyltransferase 1 (LPCAT1)	Glycerophospholipid metabolism, ether lipid metabolism
	1-Methyl-2-dodecyl-4-(1h)-quinolone	DAB adaptor protein 2	Endocytosis
	Angelicin	G protein-coupled receptor kinase 2, G protein-coupled receptor kinase 3	Endocytosis
		Ecto-5'-nucleotidase (NTE5), cytosolic II	Nicotinate and nicotinamide metabolism
	Phenylacetic acid	nicotinamide N-methyltransferase (NNMT), quinolinate phosphoribosyltransferase (QPRT)	Nicotinate and nicotinamide metabolism
	Uridine	ectonucleotide pyrophosphatase/phosphodiesterase 1, NTE5, cytosolic II	Nicotinate and nicotinamide metabolism
	1,2-Benzenedicarboxylic acid	NNMT, QPRT	Nicotinate and nicotinamide metabolism
	Choline	AChE (Cartwright blood group), phospholipase D1, phospholipase D2	Glycerophospholipid metabolism
		Phosphate cytidylyltransferase 1, choline, alpha	Phosphonate and phosphinate metabolism
		Phosphate cytidylyltransferase 1, choline, β	Ether lipid metabolism
		Phosphoethanolamine/phosphocholine phosphatase 1	Endocytosis
		Phosphate cytidylyltransferase 1, choline, β	Ether lipid metabolism
*Rehmannia glutinosa* Libosch. (prepared) (熟地黃)	Uridine	Ectonucleotide pyrophosphatase/phosphodiesterase 1, NTE5, cytosolic II	Nicotinate and nicotinamide metabolism
*Ligusticum chuanxiong* Hort. (川芎)	13-Methyl pentadecanoic acid, hexadecanoic acid, methyl pentadecanoate, pentadecanoic acid	PLA2G1B	Glycerophospholipid metabolism, ether lipid metabolism
	linoleic acid, oleic acid	LPCAT1	Glycerophospholipid metabolism, ether lipid metabolism
	Dibutyl phthalate, methyl phenylacetate, *n*-butyl-2-ethylbutylphthalate, o-cresol, *o*-ethylphenol, thymol	AChE (Cartwright blood group)	Glycerophospholipid metabolism
	Choline	AChE (Cartwright blood group), phospholipase D1, phospholipase D2	Glycerophospholipid metabolism
		Phosphate cytidylyltransferase 1, choline, alpha	Phosphonate and phosphinate metabolism
		Phosphate cytidylyltransferase 1, choline, β	Ether lipid metabolism
		Phosphoethanolamine/phosphocholine phosphatase 1	Endocytosis
		Phosphate cytidylyltransferase 1, choline, β	Ether lipid metabolism
*Eclipta prostrata* L. (旱蓮草)	Nicotine	Choline *O*-acetyltransferase	Glycerophospholipid metabolism
	Nicotinic acid	NNMT, QPRT	Nicotinate and nicotinamide metabolism, nicotinate and nicotinamide metabolism
*Lycium barbarum* L. (枸杞子)	Ascorbic acid, riboflavin, vitamin B2	Folate receptor (FR)-β, FR-γ	Endocytosis
	Safranal	AChE (Cartwright blood group)	Glycerophospholipid metabolism
	Nicotinic acid	NNMT, QPRT	Nicotinate and nicotinamide metabolism, Nicotinate and nicotinamide metabolism
*Rehmannia glutinosa* Libosch. (生地黄)	Adenosine	Purine nucleoside phosphorylase	Nicotinate and nicotinamide metabolism
	Uridine	Ectonucleotide pyrophosphatase/phosphodiesterase 1, NTE5, cytosolic II	Nicotinate and nicotinamide metabolism
*Astragalus membranaceus* (Fisch.) Bge. (黃芪)	Choline	AChE (Cartwright blood group), phospholipase D1, phospholipase D2	
		Phosphate cytidylyltransferase 1, choline, alpha	
		Phosphate cytidylyltransferase 1, choline, β	
		Phosphoethanolamine/phosphocholine phosphatase 1	
	Uridine	Ectonucleotide pyrophosphatase/phosphodiesterase 1, NTE5, cytosolic II	
*Paeonia lactiflora* Pall. (白芍藥)	Gallic acid	Diacylglycerol kinase alpha	Glycerophospholipid metabolism
*Salvia miltiorrhiza* Bge. (丹蔘)	Dauricine, Ferruginol	AChE (Cartwright blood group)	Glycerophospholipid metabolism
	dehydromiltirone, Miltirone	DAB adaptor protein 2	Endocytosis
	miltionone I, neocryptotanshinone ii, neotanshinone c, tanshiquinone b	Fibroblast growth factor receptor 2 (FGF-2)	Endocytosis
	dihydrokaranone	NTE5	Nicotinate and nicotinamide metabolism
*Morus alba* L. (桑椹)	Linoleic acid, oleic acid	LPCAT1	Glycerophospholipid metabolism, ether lipid metabolism
	Myoinositol	AChE (Cartwright blood group), phosphate cytidylyltransferase 1, choline, alpha, phospholipase D1, phospholipase D2, phosphate cytidylyltransferase 1, choline, β, phosphoethanolamine/phosphocholine phosphatase 1	Glycerophospholipid metabolism, phosphonate and phosphinate metabolism, ether lipid metabolism, endocytosis
	Trigonelline	NNMT, QPRT	Nicotinate and nicotinamide metabolism
	Vitamin B2	FR-β, FR-γ	Endocytosis
*Sesamum indicum* L. (黑芝麻)	Vitamin E	FR-β, FR-γ	Endocytosis
*Platycladus orientalis* (L.) Franco (側柏葉)	Diethyl phthalate	AChE (Cartwright blood group)	Glycerophospholipid metabolism
	Geranylacetone, verbenone	NTE5	Nicotinate and nicotinamide metabolism
	Isopimaric acid	LPCAT1	Glycerophospholipid metabolism, ether lipid metabolism
	Juniperic acid	PLA2G1B	Glycerophospholipid metabolism, ether lipid metabolism
*Gastrodia elata* Bl. (天麻)	Citronellal, dauricine	AChE (Cartwright blood group)	Glycerophospholipid metabolism
	m-Hydroxybenzoic acid	Phospholipase A2 group IIE	Glycerophospholipid metabolism, ether lipid metabolism
*Carthamus tinctorius* L. (紅花)	Arachidic acid	PLA2G1B	Glycerophospholipid metabolism, ether lipid metabolism
	Carvacrol, safranal	AChE (Cartwright blood group)	Glycerophospholipid metabolism
*Codonopsis pilosula* (Franch.) Nannf. (*唐蔘*)	13-Methyl pentadecanoic acid, azelaic acid, caprylic acid, heneicosanic acid, methyl pentadecanoate, nonadecanoic acid, octadecanoic acid, pentadecanoic acid, stearic acid	PLA2G1B	Glycerophospholipid metabolism, ether lipid metabolism
	2,4-nonadienal, nona-2,4-dienal, phenylic acid	AChE (Cartwright blood group)	Glycerophospholipid metabolism
	Stigmasta-5,22-dien-3-one, taraxerone	NTE5	Nicotinate and nicotinamide metabolism
	Alpha-curcumene	Arrestin β 2, RAB7A, member RAS oncogene family	Endocytosis
		Purine nucleoside phosphorylase	Nicotinate and nicotinamide metabolism
	Choline	AChE (Cartwright blood group), phospholipase D1, phospholipase D2	Glycerophospholipid metabolism
		Phosphate cytidylyltransferase 1, choline, alpha	Phosphonate and phosphinate metabolism
		Phosphate cytidylyltransferase 1, choline, β	Ether lipid metabolism
		Phosphoethanolamine/phosphocholine phosphatase 1	Endocytosis
	Coelogin	Diacylglycerol kinase alpha	Glycerophospholipid metabolism
	Nicotine	Choline *O*-acetyltransferase	Glycerophospholipid metabolism
	Nicotinic acid	NNMT, QPRT	Nicotinate and nicotinamide metabolism
*Chaenomeles speciosa* (Sweet) Nakai (木瓜)	2-Hexenal, diethyl phthalate	AChE (Cartwright blood group)	Glycerophospholipid metabolism
	Azelaic acid, dodecanoic acid	PLA2G1B	Glycerophospholipid metabolism, ether lipid metabolism
	Fumaric acid, linoleic acid, oleic acid, palmitoleic acid	LPCAT1	Glycerophospholipid metabolism, ether lipid
	Phenylacetic acid	NNMT, QPRT	Nicotinate and nicotinamide metabolism
	*p*-Hydroxybenzoic acid	Phospholipase A2 group IIE	Glycerophospholipid metabolism, ether lipid metabolism

**Table 3:** Target protein and associated pathways.

Target protein (KO definition)	KEGG hsa ID	Associated compound number	Associated pathway
Acetylcholinesterase (AChE) (Cartwright blood group)^a^	43	27	Glycerophospholipid metabolism, cholinergic synapse
PLA2G1B	5319	14	Glycerophospholipid metabolism, ether lipid metabolism arachidonic acid metabolism, linoleic acid metabolism, alpha-Linolenic acid metabolism, metabolic pathways, RAS signaling pathway, vascular smooth muscle contraction, pancreatic secretion, fat digestion and absorption
LPCAT1	79,888	6	Glycerophospholipid metabolism, ether lipid metabolism, metabolic pathways
Ecto-5'-nucleotidase (NTE5)	4907	5	Purine metabolism, pyrimidine metabolism, nicotinate and nicotinamide metabolism, metabolic pathways
Fibroblast growth factor receptor 2 (FGF-2)^a^	2263	4	EGFR tyrosine kinase inhibitor resistance, MAPK signaling pathway, RAS signaling pathway, Rap1 signaling pathway, endocytosis, PI3K-Akt signaling pathway, signaling pathways regulating pluripotency of stem cells, regulation of actin cytoskeleton, pathways in cancer, prostate cancer, gastric cancer, central carbon metabolism in cancer
FR-β	2350	4	Antifolate resistance, endocytosis
FR-γ	2352	4	Antifolate resistance, endocytosis
NNMT	4837	4	Nicotinate and nicotinamide metabolism, metabolic pathways
QPRT	23,475	4	Nicotinate and nicotinamide metabolism, metabolic pathways
DAB adaptor protein 2	1601	3	Endocytosis
Diacylglycerol kinase alpha	1606	2	Glycerolipid metabolism, glycerophospholipid metabolism, metabolic pathways, phosphatidylinositol signaling system, phospholipase D signaling pathway, choline metabolism in cancer
Purine nucleoside phosphorylase	4860	2	Purine metabolism, pyrimidine metabolism, nicotinate and nicotinamide metabolism, metabolic pathways
Phosphate cytidylyltransferase 1, choline, alpha	5130	2	Phosphonate and phosphinate metabolism, glycerophospholipid metabolism, metabolic pathways, choline metabolism in cancer
Phospholipase D1	5337	2	Glycerophospholipid metabolism, ether lipid metabolism, metabolic pathways, RAS signaling pathway, cAMP signaling pathway, sphingolipid signaling pathway, phospholipase D signaling pathway, endocytosis, Fc γ R-mediated phagocytosis, glutamatergic synapse, gonadotropin-releasing hormone (GnRH) signaling pathway, parathyroid hormone synthesis/secretion/action, pathways in cancer, pancreatic cancer, choline metabolism in cancer
Phospholipase D2	5338	2	Glycerophospholipid metabolism, ether lipid metabolism, metabolic pathways, RAS signaling pathway, cAMP signaling pathway, sphingolipid signaling pathway, phospholipase D signaling pathway, endocytosis, Fc γ gamma R-mediated phagocytosis, glutamatergic synapse, GnRH signaling pathway, parathyroid hormone synthesis, secretion and action, pathways in cancer, pancreatic cancer, choline metabolism in cancer
Phosphate cytidylyltransferase 1, choline, β	9468	2	Phosphonate and phosphinate metabolism, glycerophospholipid metabolism, metabolic pathways, choline metabolism in cancer
NTE5	22,978	2	Purine metabolism, pyrimidine metabolism, nicotinate and nicotinamide metabolism, metabolic pathways
Phospholipase A2 group IIE	30,814	2	Glycerophospholipid metabolism, ether lipid metabolism, arachidonic acid metabolism, linoleic acid metabolism, alpha-linolenic acid metabolism, metabolic pathways, RAS signaling pathway, vascular smooth muscle contraction, pancreatic secretion, fat digestion and absorption
Phosphoethanolamine/phosphocholine phosphatase 1	162,466	2	Glycerophospholipid metabolism, metabolic pathways
G protein-coupled receptor kinase 3	157	1	Chemokine signaling pathway, endocytosis, Hedgehog signaling pathway, glutamatergic synapse, olfactory transduction, morphine addiction
G protein-coupled receptor kinase 2	156	1	Chemokine signaling pathway, endocytosis, Hedgehog signaling pathway, glutamatergic synapse, olfactory transduction, morphine addiction
Arrestin β 2	409	1	MAPK signaling pathway, chemokine signaling pathway, endocytosis, Hedgehog signaling pathway, dopaminergic synapse, olfactory transduction, Relaxin signaling pathway, parathyroid hormone synthesis, secretion and action, GnRH secretion, morphine addiction
Choline O-acetyltransferase	1103	1	Glycerophospholipid metabolism, cholinergic synapse
RAB7A, member RAS oncogene family	7879	1	Mitophagy–animal, autophagy–animal, endocytosis, phagosome, *Salmonella* infection, amoebiasis, tuberculosis
Ectonucleotide pyrophosphatase/phosphodiesterase 1	5167	1	Purine metabolism, pyrimidine metabolism, starch and sucrose metabolism, riboflavin metabolism, nicotinate and nicotinamide metabolism, pantothenate and CoA biosynthesis, metabolic pathways

Sorted by the number of associated compounds of the target protein; hsa, *Homo sapiens.*

^a^Target proteins overlapping with the Genecards database search results, i.e., AChE and FGF-2 overlapped with results when ‘alopecia’ was used as a disease keyword in the Genecards database.

**Table 4:** Associated pathways of 20 vital medicinal herbs.

Associated pathway	Frequency
Metabolic pathways	15
Glycerophospholipid metabolism	11
Endocytosis	10
Nicotinate and nicotinamide metabolism	6
Choline metabolism in cancer	5
Ether lipid metabolism	5
RAS signaling pathway	5
Glutamatergic synapse	4
Purine metabolism	4
Pyrimidine metabolism	4
Chemokine signaling pathway	3
Hedgehog signaling pathway	3
Morphine addiction	3
Olfactory transduction	3
Parathyroid hormone synthesis, secretion, and action	3
Pathways in cancer	3
Phospholipase D signaling pathway	3
Alpha-linolenic acid metabolism	2
Antifolate resistance	2
Arachidonic acid metabolism	2
cAMP signaling pathway	2
Cholinergic synapse	2
Fat digestion and absorption	2
Fc γ R-mediated phagocytosis	2
GnRH signaling pathway	2
Linoleic acid metabolism	2
MAPK signaling pathway	2
Pancreatic cancer	2
Pancreatic secretion	2
Phosphonate and phosphinate metabolism	2
Sphingolipid signaling pathway	2
Vascular smooth muscle contraction	2
Amoebiasis	1
Autophagy-animal	1
Central carbon metabolism in cancer	1
Dopaminergic synapse	1
EGFR tyrosine kinase inhibitor resistance	1
Gastric cancer	1
Glycerolipid metabolism	1
GnRH secretion	1
Mitophagy—animal	1
Pantothenate and CoA biosynthesis	1
Phagosome	1
Phosphatidylinositol signaling system	1
PI3K-Akt signaling pathway	1
Prostate cancer	1
Rap1 signaling pathway	1
Regulation of actin cytoskeleton	1
Relaxin signaling pathway	1
Riboflavin metabolism	1
Salmonella infection	1
Signaling pathways regulating pluripotency of stem cells	1
Starch and sucrose metabolism	1
Tuberculosis	1

Sorted by the frequency of pathways presented in Table 3.

*GnRH* gonadotropin-releasing hormone.

## Results

### Extraction of information on the included compounds in 20 vital medicinal herbs using TCMID data

The compounds included in 20 vital medicinal herbs are shown in Supplementary Material 1.

### Construction of the herb-compound (ingredient)-associated target protein-associated target pathway network

Based on the predefined OB and DL criteria, we explored the TCMID database and extracted information on 77 potential compounds isolated from 20 medicinal herbs. Based on the predefined prediction score, a further search using the BATMAN-TCM database resulted in the extraction of information on 25 associated potential target genes from 77 potential compounds. From the proteins targets, information on 54 associated potential target pathways was extracted from the KEGG pathway database, which presented with a statistically higher weighted degree of centrality than that obtained using the null model. The tripartite network comprising 77 compounds, 25 target proteins, and 54 target pathways of 20 vital medicinal herbs are presented in Table 2 in an alphabetical order of the ingredients for the convenience of the readers. Detailed results regarding the weighted degree of centrality have been presented in Supplementary Material 2.

Twenty-five target proteins of potential compounds and fifty-four associated target pathways are presented in Table 3, sorted by the number of target protein-associated compounds. Acetylcholinesterase (AChE), phospholipase A2 group IB (PLA2G1B), and lysophosphatidylcholine acyltransferase 1 (LPCAT1) were associated with 27, 14, and 6 potential compounds, respectively. Particularly, AChE and fibroblast growth factor receptor 2 (FGF-2) among the findings overlapped with the target protein derived when alopecia was explored as a disease keyword in the GeneCards database.

The 54 associated pathways of 20 vital medicinal herbs are presented in Table 4, sorted by the frequency of pathways presented in Table 3**.** The sequence in Table 4 indicates the major possible mechanism of THM-based alopecia treatment. The most frequently associated target pathway is the metabolic pathway (frequency 15), followed by glycerophospholipid metabolism (frequency 11), endocytosis (frequency 10), and nicotinate and nicotinamide metabolism pathways (frequency 6).

### Visualization of the herb-potential compound (ingredient)-associated gene network

**Figure 2:**
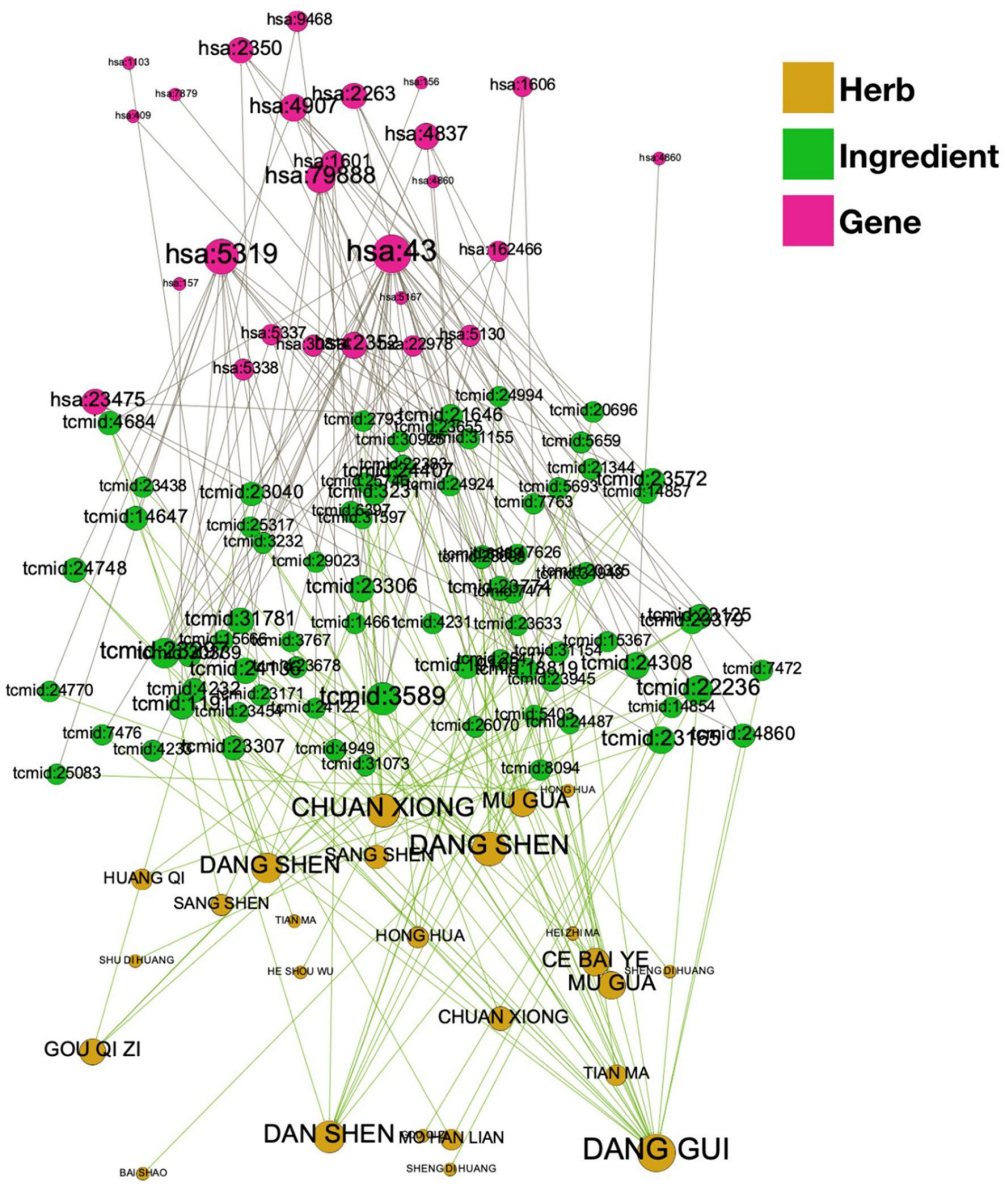
Herb–compound–gene network.

We have constructed and presented a herb-compound (ingredient)-associated gene network (Fig. 2), and a larger node indicates herbs/ingredients (compound)/genes to a greater degree. AChE, hsa43 in KEGG hsa ID), and PLA2G1B (5319 in KEGG hsa ID) were associated with 27 and 14 potential compounds, respectively, and have been indicated with the two largest gene nodes in Fig. 2.

## Discussion

### Summary of findings

In this study, we explored the possible mechanisms of action of 20 vital medicinal herbs for alopecia treatment using a network pharmacology analysis method for the first time. We extracted information on 77 potential compounds isolated from the 20 main medicinal herbs, and 25 potential target proteins/genes (from the 77 compounds) were found to be associated with 54 target pathways. AChE and PLA2G1B represented the largest nodes in the potential protein (gene) group. Metabolic, glycerophospholipid, and endocytosis pathways were the most frequently associated pathways for the mechanism of action of 20 vital medicinal herbs.

The potential mechanism of action of 20 vital medicinal herbs for alopecia treatment may mainly be mediated via regulation of various target genes/proteins and target pathways, including glycerophospholipid metabolism in cholinergic synapses driven by acetylcholinesterase, lipid metabolism driven by various phospholipase PLA2 subtypes, the purine pathway driven by ecto-5-nucleotidase (NTE5) in the hair follicle cycle, macrophage-mediated inflammation via regulation of folate receptors (FRs), NNMT, and QPRT linking NAD + expression and activity, or DAB adaptor protein 2 (DAB2) inhibition of Wnt signaling.

### Main target proteins with associated pathways derived from 20 vital medicinal herbs for alopecia treatment

In a previous study, we investigated the modular characteristics of medicinal herbs for alopecia treatment in TCM using network analysis and extracted information on 20 main medicinal herbs for alopecia treatment. These herbs are closely related to the “liver” and “kidney” function as per the TCM theory. The liver and kidneys play important roles in lipid metabolism. The liver is responsible for overall fatty acid synthesis and lipid circulation^28^, and the kidney possesses a high absorption capacity for lipid-binding proteins and lipid-regulating hormones^29^. Lipids are essential components of cellular membranes that act as skin barriers and demonstrate functions as bioactive lipid mediators^30^.

In our study, we found that the main components of the 20 main medicinal herbs were closely associated with pivotal target proteins, including acetylcholinesterase (27 compounds), PLA2G1B (14 compounds), LPCAT1 (six compounds), NTE5 (five componds), fibroblast growth factor receptor 2 (FGF-2, four compounds), FR-β and -γ (four compounds each), NNMT (four compounds), QPRT (four compounds), and DAB adaptor protein 2 (three compounds). These target proteins play a key role in mediating the therapeutic effects of medicinal herbs on alopecia by regulating the main pathways, including glycerophospholipid metabolism, choline metabolism, endocytosis, nicotinate/nicotinamide metabolism, ether lipid metabolism, RAS signaling pathway, glutamatergic synapse, purine metabolism, and pyrimidine metabolism involved in the hair follicle cycle.

After comparing the data for the hair loss target protein extracted using the Genecard database with those of the target protein of herbal medicine for hair loss, only AChE and FGF-2 were found to overlap, whereas the other target proteins did not present with overlapping. These findings indicate that medicinal herbs used for alopecia treatment can act on target proteins, such as AChE and FGF-2, and demonstrate association with alopecia; however, they can exert a therapeutic effect on alopecia via the expression of distinct target genes, proteins, and pathways compared to those observed in conventional medicine. The findings reported in this study based on medicinal herbs that may be used for alopecia treatment can help provide insights into a new potential therapeutic target that may be distinct from the one reported in conventional medicine.

#### Target proteins of medicinal herbs for alopecia treatment showing an overlap with alopecia target proteins in the Genecards database

##### AChE

In our study, AChE was frequently associated with 27 ingredients in the 20 vital medicinal herbs. In cholinergic synapses, AChE is associated with glycerophospholipid metabolism and cholinergic synapses (Table 3). AChE catalyzes the hydrolysis of the neurotransmitter acetylcholine (ACh), a component which is responsible for the exhibition of its anti-inflammatory properties^31^. Cholinergic neurons release ACh to induce chemical transmission via ACh nicotinic and muscarinic receptors^32^. ACh receptors play an important role in controlling the hair follicle cycle^33^.

Neuronal or non-neuronal ACh signaling or both lead to the depletion of stem cell populations in murine hair follicles in a complex neuroectodermal–mesodermal interaction system^32^. AChE has been reported to induce hair growth^34^. The glycerophospholipid metabolic pathway maintains cell membrane stability to confer protection to the cell against hypoxic stress-induced damage by upregulating mRNA, protein, and metabolite levels^35^. Therefore, the main medicinal herbs may affect alopecia by modulating AChE to regulate ACh signaling in hair follicle stem cells and to regulate the glycerophospholipid metabolic pathway. We suggest that AChE may be a key target active protein in medicinal herbs for hair loss treatment.

##### FGF-2

Growth factors, including FGF, insulin-like growth factor (IGF)-1, vascular endothelial growth factor (VEGF), and epidermal growth factor (EGF), regulate hair morphogenesis and the hair cycle^36,37^ Particularly, FGF-2 is known to activate dermal papilla cell proliferation and to increase the size of hair follicles^37^. In our study, FGF-2 was identified as one of the major target proteins of main medicinal herbs for alopecia treatment. An experimental study showed that topical application of FGFs, including FGF-2, induced the anagen phase in telogenic C57BL/6 mice^36^. Adenosine stimulates the growth of the dermal papilla and prolongs the anagen phase by upregulating the cysteine levels modulated by FGF-2 and FGF-7^36^. Therefore, the therapeutic effect of the main medicinal herbs on alopecia may involve the modulation of FGF-2 to promote dermal papilla cell proliferation and to prolong the anagen phase by increasing cysteine levels.

#### Potential target proteins of medicinal herbs for alopecia treatment not showing an overlap with alopecia target proteins in the Genecards database

##### LPCAT1 and PLA2 family (secretory PLA2IB [sPLA2GIB] and PLA2G IIE)

Lysophosphatidylcholine (LPC) is a class of lipid biomolecules derived from the cleavage of phosphatidylcholine (PC) via the action of PLA2, the transfer of fatty acids to free cholesterol via LPCAT, or both^38^. PLA2 family members were identified as important target proteins in our study (Table 3). The secretory PLA2 (sPLA2) family exhibits unique tissue and cellular distributions and enzymatic properties, including production of pro- and anti-inflammatory lipid mediators, regulation of membrane remodeling, and modification of extracellular noncellular lipid components^39^. The PLA2 family affects cutaneous homeostasis via the supply of fatty acids and lysophospholipids and regulates skin barrier function^30^. Particularly, sPLA2IIE is involved in metabolic regulation and hair follicle homeostasis. sPLA2IIE is expressed abundantly in hair follicles in synchrony with the hair growth cycle. sPLA2IIE is also distributed in companion cells of the outer root sheath and cuticular cells of the inner root sheath, but its expression is immediately downregulated to a negligible level during the catagen to telogen phase, and is subsequently upregulated when the cells enter the next anagen phase^30^. sPLA2GIB has been reported to be involved in cell proliferation, cell migration, hormone release, and eicosanoid production in peripheral tissues through actions mediated by its receptors^40^. In our study, sPLA2GIB was identified as one of the major target proteins from the 20 medicinal herbs of Module 1 for alopecia treatment. However, related studies remain relatively insufficient and, therefore, further research is warranted to elucidate the contributory role of sPLA2GIB in hair growth.

##### NTE5 (CD73)

NT5E (CD73) is an enzyme expressed on free nerve endings in the epidermis and skin cells, which regulates purinergic signaling by desphosphorylating extracellular AMP to adenosine in nociceptive circuits^41^. High levels of NT5E (CD73) have also been found in hair follicle cells^42^. Dermal microcirculation is essential for supplying various growth factors and other bioactive molecules for hair maintenance^43^. Particularly, during the anagen phase, such a mechanism of dermal microcirculation is important for maintaining the high metabolic activity of hair follicle matrix cells.

The mechanism of action of MXD, a topical application for alopecia, has not been completely elucidated. However, MXD is known to induce the release of adenosine in follicular keratinocytes and to promote the release of vascular endothelial growth factors in dermal papilla cells, thereby promoting dermal microcirculation^43^. In our study, NT5E (CD73) was one identified as of the major target proteins of the medicinal herbs for alopecia treatment. Therefore, 20 vital medicinal herbs for alopecia treatment may act by modulating NT5E (CD73) to promote dermal microcirculation through the regulation of purinergic signaling by desphosphorylating extracellular AMP to adenosine in hair follicles and dermal papilla cells.

##### FR

In our study, FR-β and -γ were associated with four ingredients in 20 main medicinal herbs for alopecia treatment. The FR, which presents with three isoforms, namely FR-α, FR-β, and FR-γ in humans, is a cell surface glycosylphosphatidylinositol (GPI)-anchored glycoprotein with a high affinity for folic acid^44^. A previous study has shown that FR-β is highly expressed on activated macrophages, cells which are found in various activated macrophage-mediated inflammatory diseases, including rheumatoid arthritis, psoriasis, Crohn's disease, and systemic lupus erythematosus^44^. Dermis-resident TREM2 + macrophages reportedly promote hair follicle stem cell quiescence and help maintain telogen via the regulation of Janus kinase (JAK)-signal transducer and activator of transcription 5 (STAT5) signaling^45^. Conversely, regulatory T cells are known to promote hair follicle stem cell activation and hair regrowth^45^. Therefore, the therapeutic effects of the vital medicinal herbs on alopecia may be involved in macrophage-mediated inflammation through the regulation of FRs, especially FR-β.

##### NNMT and QPRT

NNMT is a metabolic enzyme that catalyzes the methylation of nicotinamide to enable the formation of N-methylnicotinamide using the universal methyl donor *S*-adenosyl methionine (SAM), which directly links one-carbon metabolism with the methylation balance and nicotinamide adenine dinucleotide (NAD +) levels of cells as therapeutic targets in addition to their metabolic function in detoxification^46,47^. NNMT activity and expression are modulated differently in various tissues. While NNMT in the adipose tissue is involved in obesity and insulin resistance, its expression in the liver demonstrates beneficial effects by regulating lipid parameters^47^. In the present study, the main medicinal herbs for alopecia treatment correlated closely with the liver and kidney as per the TCM theory. Although evidence to support the direct relationship between NNMT and alopecia is lacking, the vital medicinal herbs for alopecia treatment may exert a therapeutic effect by controlling lipid parameters in the liver.

QPRT is a key enzyme involved in the de novo synthesis of NAD + that presents with a restricted tissue distribution, including in the liver and kidney, which is involved in the effects of 20 main medicinal herbs for alopecia treatment in our study^48^. NAD + is synthesized via de novo synthesis, and the salvage pathway is an essential cofactor of the oxidation–reduction reaction^48^. NAD + activity is associated with various cellular functions, including calcium homeostasis, antioxidant activity, gene expression, and apoptosis^49^.

An experimental study has suggested that QPRT acts as a suppressor of spontaneous cell death by inhibiting the overproduction of active caspase 3^48^. 20 vital medicinal herbs that affect liver function as per the TCM theory may act on alopecia by regulating NNMT and QPRT. Our findings suggest that NNMT and QPRT, which link NAD + expression and activity, may be deemed potential therapeutic targets for alopecia treatment.

##### DAB2

In this study, DAB2 was identified as a target protein associated with three compounds involved in 20 main medicinal herbs for alopecia treatment. DAB2 is a cargo-binding endocytic adaptor protein that controls cellular homeostasis and is implicated in several receptor-mediated signaling pathways, cell adhesive function, hematopoietic cell differentiation, and angiogenesis^50^. Additionally, DAB2 plays a vital role in regulating adipocyte cell size, number, and T cell function in the immune system^51^. Several studies have reported that DAB2 inhibits canonical Wnt signaling^52^ and plays a pivotal role in the initiation of hair follicle placode formation and development^53,54^. Androgen is known to downregulate the expression of dermal papilla cell-secreted factors associated with hair follicle stem cell differentiation through the inhibition of canonical Wnt signaling^55^. Therefore, the main medicinal herbs may exert an effect on alopecia via modulation of DAB2, leading to the inhibition of Wnt signaling.

### Limitations, significance, and suggestions for further studies

This study presents with several limitations. First, only the BATMAN-TCM^19^ and TCM-ID^18^ databases were considered. However, these are databases that are widely considered in TCM network pharmacology research. Thus, we focused on the database specified in the TCM. Although there is a disadvantage of limited information in the database, a drawback due to which the search results may be omitted, it is already known that TCM-related databases present with high redundancy when information on medicinal herbs is explored. Moreover, search terms for medicinal herbs in the database must be narrowed for the TCM network pharmacologic research, as many medicinal herbs have different aliases in general databases^56^. Therefore, despite several shortcomings, a TCM-specific database was selected. In future studies, it is therefore recommended to use a more extensive database. Second, Presently, molecular docking methods are utilized to explore and visualize the interaction between the candidate target and the compound^57^. Even though we did not adopt molecular docking analysis in the present study, such an approach along with experimental study in further studies may deepen the understanding of the mechanism. In addition, a draft for method evaluation guidelines for network pharmacology is under development^58^. In future studies, expectedly, a more rigorous research methodology will be adopted using the developed guideline^58^. Finally, our results should be verified as these findings suggest a probable and possible mechanism via a network pharmacology analysis approach based on TCM medicinal herbs.

Despite such limitations, we confirm that this is a novel approach to use a network pharmacologic method to investigate the effect of medicinal herbs on the treatment of alopecia. Selection of medicinal herbs was performed based on the information presented in previous studies and network analyses. Here, exploration of a possible mechanism of medicinal herbs, which is different from a conventional alopecia drug mechanism, has been implemented. Our findings using a network pharmacological approach may help better understand the system-level mechanisms of action of multi-component and multi-target THM preparations for alopecia treatment. However, we anticipate that the mechanism of action of 20 vital medicinal herbs against alopecia may be associated with complex pathways and warrants further proof of concept of the target genes/proteins and target pathways suggested by our research. This is because network pharmacology is only a predictive tool.

## Conclusions

In this study, we analyzed a novel possible mechanism of action of 20 main medicinal herbs for alopecia treatment using a network pharmacological approach. We also determined the medicinal herb-ingredient-target protein network and constructed an ingredient-associated protein (gene)-associated pathway network. In conjunction, our data suggest that the effects of the vital medicinal herbs for alopecia treatment may be mediated mainly through the regulation of various target genes/proteins including AchE, PLA2 subtypes, NTE5, FR, NNMT, QPRT, and DAB2, and target pathways including glycerophospholipid metabolism, choline metabolism, endocytosis, nicotinate/nicotinamide metabolism, ether lipid metabolism, RAS signaling pathway, glutamatergic synapse, purine metabolism, and pyrimidine metabolism involved in the hair follicle cycle.

These findings regarding target genes/proteins and target pathways of vital medicinal herbs associated with alopecia treatment will provide a novel foundation and will help support further studies to enhance our understanding of the therapeutic mechanism of medicinal herbs for alopecia treatment in TCM and to further elucidate the pathogenesis of alopecia.

## Supplementary Information

Supplementary Information 1.

Supplementary Information 2.

## Data Availability

The datasets used or analyzed during the current study will be available from the corresponding author upon reasonable request.

## Abbreviations

AChE: Acetylcholinesterase
ADME: Absorption, distribution, metabolism, and elimination
BATMAN-TCM: Bioinformatics analysis tool for molecular mechanism of traditional Chinese medicine
CAM: Complementary and alternative medicine
DAB2: DAB adaptor protein 2
DL: Drug-likeness index
FGF-2: Fibroblast growth factor 2
FR: Folate receptor
GO: Gene ontology
KEGG: Kyoto encyclopedia of genes and genomes
FDR: False discovery rate
OB: Oral bioavailability
LPCAT1: Lysophosphatidylcholine acyltransferase 1
NNMT: Nicotinamide *N*-methyltransferase
MXD: Minoxidil
NTE5: Ecto-5-nucleotidase
PLA2: Phospholipase A2
QPRT: Quinolinate phosphoribosyltransferase
sPLA2GIB: Secretory phospholipase A2 group IB
TCM: Traditional Chinese medicine
TCMID: Traditional Chinese medicine integrated database
THM: Traditional herbal medicine
