# Supplementary material for: A network pharmacology-based approach to explore mechanism of action of medicinal herbs for alopecia treatment
Source: Sci Rep. 2022 Feb 18;12:2852. doi: 10.1038/s41598-022-06811-6 (PMC8857194; doi:10.1038/s41598-022-06811-6)
Supplement: Supplementary file 1 — Supplementary Information 1. [file 41598_2022_6811_MOESM1_ESM.pdf]

	Unnamed: 0	cn_name	eng_name	Ingredients	Ingredient_indexes	Number of included ingredien	latin_name	py_name
0	1344	党参	Pilose Asiabell Equivalent plant: Codonopsis pilosula var modesta, Codonopsis tangshen, Codonopsis tubulosa, Codonopsis subglobosa, Codonopsis canescens, Codonopsis clematidea	[illegible]	[illegible]	187	Codonopsis pilosula	DANG SHEN
1	2473	红花	Safflower	[illegible]	[illegible]	71	Carthamus tinctorius	HONG HUA
2	2537	当归	Chinese Angelica Equivalent plant: Phlojodicarpus sibiricus	[illegible]	[illegible]	163	Angelica sinensis	DANG GUI
3	2566	白芍	Common Peony	[illegible]	[illegible]	54	Paeonia albiflora [Syn. Paeonia lactiflora]	BAI SHAO
4	4255	生地黄	Fresh rehmannia root	[illegible]	[illegible]	49	Radix Rehmanniae	SHENG DI HUANG
5	4271	侧柏叶	Chinese Arborvitae Leaf	[illegible]	[illegible]	137	Thuja orientalis	CE BAI YE
6	4888	木瓜	Chinese Floweringquince	[illegible]	[illegible]	172	Chaenomeles sinensis	MU GUA
7	5403	菟丝子	Dodder Seed	[illegible]	[illegible]	18	Semen Cuscutae;Semen Cuscutae	TU SI ZI
8	5893	熟地黄	Adhesive Rehmannia Cocked Root	[illegible]	[illegible]	13	Rehmannia glutinosa [Syn. Rehmannia glutinosa [illegible]	SHU DI HUANG
9	5925	川芎	Chuanxiong (Wallich Ligusticum) Equivalent plant: Chidium officinale	[illegible]	[illegible]	182	Ligusticum chuanxiong [Syn. Ligusticum wallichii]	CHUAN XIONG
[illegible]	6278	何首乌	Tuber Fleeceflower	[illegible]	[illegible]	72	Polygonum multiflorum	HE SHOU WU
[illegible]	6648	女贞子	fruit of Glossy privet	[illegible]	[illegible]	18	Fructus Ligustri lucidi	NU ZHEN ZI
[illegible]	6738	天麻	Tall Gastrodia	[illegible]	[illegible]	46	Gastrodia elata	TIAN MA
[illegible]	[illegible]	[illegible]	Oriental Sesame (black seed)	[illegible]	[illegible]	[illegible]	[illegible]	[illegible]
[illegible]	6851	黄精	Siberian Solomonseal Equivalent plant: Polygonatum kingianum, Polygonatum cyrtonema	[illegible]	[illegible]	16	Polygonatum sibiricum	HUANG JING
[illegible]	6916	黄芪	Membranous Milkvetch Equivalent plant: Astragalus mongholicus, Astragalus chrysopterus, Astragalus ernestii	[illegible]	[illegible]	70	Astragalus membranaceus	HUANG QI
[illegible]	6952	墨旱莲	Yerbadetajo	[illegible]	[illegible]	37	Eclipta prostrata [Syn. Eclipta alba]	MO HAN LIAN
[illegible]	7293	丹参	Danshen Equivalent plant: Salvia przewalskii, Salvia miltiorrhiza f. alba	[illegible]	[illegible]	166	Salvia miltiorrhiza	DAN SHEN
[illegible]	7295	桑椹	Mulberry Fruit	[illegible]	[illegible]	85	Fructus Mori	SANG SHEN
[illegible]	7304	枸杞子	Chinese Wolfberry Fruit Equivalent plant: Lycium barbarum	[illegible]	[illegible]	102	Lycium chinense	GOU QI ZI
